# Cortical layer with no known function

**DOI:** 10.1111/ejn.13978

**Published:** 2018-08-03

**Authors:** Zoltán Molnár

**Affiliations:** ^1^ Department of Physiology, Anatomy and Genetics University of Oxford Oxford UK

## Introduction

I am presenting some personal accounts of my interactions with Ray Guillery during his time in Oxford, from 1988 to 1996 and then from 2000 to 2017. This essay provides specific examples of how my scientific interactions with Ray influenced my way of thinking about brain structure and function, how it helped me to develop my scientific rigour and stimulated ideas about thalamocortical development. I shall also review some of the key contributions that Ray Guillery made to the field of thalamocortical organization and interactions and how his insight is providing some guidelines for our work today. Members of my laboratory recently identified intracortical and thalamic projections from a subpopulation of layer 6b cells that might regulate both cortical and thalamic arousal of cortical areas that are involved in higher cortical functions (Hoerder‐Suabedissen, Hayashi et al., 2018). It was Ray who referred to this cortical layer in our discussions as “a layer with no known function” and encouraged us to continue its investigation.

## Anatomists and physiologist

I met Ray Guillery in 1988 when I arrived in Oxford as a Soros–Hungarian Academy of Sciences Scholar to work in Colin Blakemore's laboratory at the University Laboratory of Physiology, University of Oxford. Our encounters began in meetings and seminars, when the very strong visual neuroscience community of Oxford met in one of the departmental seminars, either in Human Anatomy, University Laboratory of Physiology, Pharmacology or Psychology. Later, when I knew Ray better, I would pop over to the Human Anatomy Department to have chats with him whilst he had tea at the common room, which incidentally was originally the mortuary directly below the large lecture theatre. Ray always had his tea at the same time of the day and sat at the end of a row of green leather armchairs, just right of the serving counter. So I always knew where to find him.

It was great to hear his views and some alternative perspectives on my often rudimentary but very enthusiastic ideas. Anatomists and physiologists were very different species; anatomists came in early, worked hard until 5 p.m. and left for home. Physiologists had a later start, spent much time generating and analysing computerized data and they stayed very late. I felt comfortable with both groups. My interactions with Ray became more frequent when I changed the topic of my research. Initially, Colin teamed me up with Jürgen Engelage to study complex receptive fields in the upper layers of the visual cortex. Jürgen was a great guy and the topic was interesting, but it involved long experiments and even longer generation of computer signals and analysis of the data. This should not have been the problem—in fact, I loved experiments. The issue was that Jürgen liked to smoke his pipe in the laboratory during experiments and I just could not handle the smoke. I smoked a pack of cigarettes at the age of six, with the permission of my parents, and I never smoked again! So when Colin suggested that I set up some basic research on thalamocortical interactions in co‐culture system based on the work of Keisuke Toyama and Nobuhiko Yamamoto (Yamamoto, Kurotani & Toyama, 1989) I jumped at the opportunity. This project sparked my interest in the early development of thalamocortical projections (Blakemore & Molnár, 1990; Molnár & Blakemore, 1991, 1995) and I decided to start some more detailed studies using carbocyanine dye tracing (Molnár, Adams & Blakemore, 1998; Molnár, Adams, Goffinet & Blakemore, 1998). Ian Thompson recommended that I talked to Ray's group to get going, so I spoke to Ray Colello, Gary Baker, Ben Reese, John Mitrofanis, Hector Chan, Joachim Lübke, John Crabtree and Jeremy Taylor about the best methods to trace various pathways of the developing visual system. They were a superb bunch and gave me a lot of help. Later, I started attending some of Ray's laboratory meetings.

## The white sheep on the hill

I eventually started attending some of Ray's laboratory meetings that happened every Friday. His meetings were held in his spacious office on the first floor of the Le Gros Clark Human Anatomy Building. They started with a presentation that could be interrupted at any time and we discussed various aspects for protracted periods. Sometimes these dragged on late into the afternoon and the team moved on to one of the local pubs to calm down after the often‐heated scientific exchanges and arguments. This is how I started hanging out with members of Ray's group, in their preferred pubs: the Royal Oak and the Lamb and Flag. Physiologists were usually at the King's Arms on Friday afternoons, Colin paying for the first round no matter how big the group was.

One of the reasons why Ray's laboratory meetings lasted so long was because all the bits of evidence were questioned at every step: data in the published literature, the presenter's experimental evidence, possible methodological pitfalls and their interpretations. Initially, I did not understand why these arguments were so entailed and critical. Sometimes we could not get through someone's introduction because some of the illustrations were not accurate or there were too many generalizations in the opening sentences, or the big picture was not sufficiently big enough. I only presented two or perhaps three times on one of these meetings and they were all very memorable. After one of my presentations where my “feathers were ruffled,” Ray Collello gave me some advice on how to present at Ray's laboratory meeting. “Imagine that you can see a bunch of white sheep grazing on a hill. You can't just say that these sheep are white. What you should say is that the profiles of the sheep show us from our current viewpoint are white.” Anything else would be undue generalization in Ray's laboratory meetings. I appreciated this advice even more when I had to re‐write some of my thesis after Ray had examined it together with Ray Lund. Instead of changing the wording, I provided the explicit evidence for almost all of the statements I made. This was an experience that helped me to develop scientific rigour and precision. This made it take much longer to finish it, but I was proud of the outcome at the end and it certainly helped my thesis win the Rolleston Memorial Prize of Oxford and Cambridge Universities for 1994 (Molnár, 1994) and later also being published it as a book with Springer (Molnár, 1998).

## Key to college

Ray was a fellow of Hertford College, because he held the Dr Lee's Chair of Human Anatomy that is historically associated with this college. Coincidentally, this was also my own college during my graduate studies. When I was awarded a Senior Scholarship from Hertford, which included having dining rights to eat on “high table” one evening a week, I was looking forward to chatting to Ray during these dinners. However, I found out that Ray hardly ever came in to dinners at college. I asked him the reasons and he told me that he just did not feel at home there and did not even ask for a key to the senior common room, so he could read the newspapers or have a tea. On hearing this, I went to see the bursar and requested a key on his behalf and I dropped it off to his department in an envelope. He then came to college a few times, not too often, and we had some interesting discussions about research and even talked about the European Journal of Neuroscience, the journal of which he was founding editor. Ray was very enthusiastic about this new journal. He loved the scientific interactions with authors and referees as the Editor. He adored the process of scientific exchanges to improve the papers. And the EJN had a very good start (Guillery, 2015). Once, Ray told me that he wanted to have the same cover image for all issues. I did not agree, since I usually consider it an honour to have your figure on the cover of a journal, but Ray considered it as an unnecessary step in publishing.

## Men in the machine

Ray and I had many detailed discussions about early thalamocortical development. Ray had shown that the sensory periphery and the central thalamocortical and corticothalamic pathways started their development separately and they only interacted at later stages (Mitrofanis & Guillery, 1993; see papers by Lamantia, 2018 and Walsh, 2018 in this issue). Therefore, the early stages of thalamocortical development take place autonomously, with the sensory periphery plugging into these immature circuits before beginning to transmit spontaneous and later sensory information. Ray was very interested in learning more about these early stages by looking at the tracings I had been doing with carbocyanine dyes in fixed tissue (Molnár, Adams & Blakemore, 1998; Molnár, Adams, Goffinet, et al., 1998). He was particularly interested in the early topography of the projections whilst they arrived at the cortex and subsequently how they spread within the cortex. I had relatively simple views on the early topography of the thalamocortical projections, but he and John Crabtree drew my attention to Simon LeVay's paper on the reorganization of the visual projection close to the primary visual cortex (Nelson & LeVay, 1985). This work involved paired tracer injections and demonstrated that the fibres in the thalamocortical projection followed a neatly organized trajectory in the optic radiation, but then changed organization a few hundred microns before they reached the cortex. Ray predicted that there had to be some kind of developmental interaction between thalamic projections and the cortex as thalamic fibres approached and accumulated there closer to the cortex. Earlier tracing studies showed that there were waiting periods that took place between transitions and studies on the subplate begun to demonstrate the substantial rearrangements of the fibres during development (Kostovic & Rakic, 1980, 1990; Shatz et al., 1990). Ray wrote a commentary on the subplate in a “News and Views” article in Nature (Guillery & Killackey, 1987).

**Figure 1:**
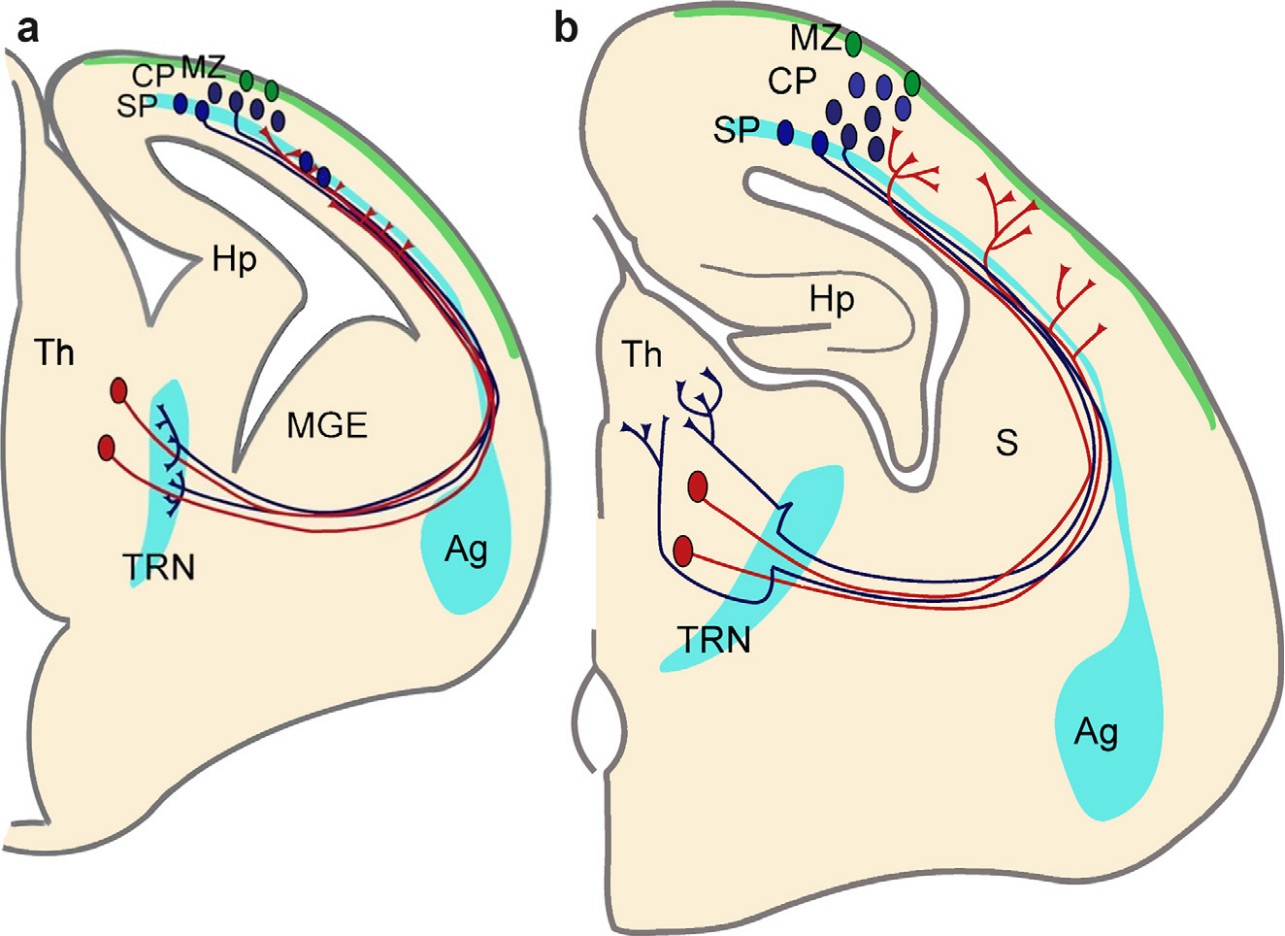
Functional correlation between the developing thalamocortical projections, cortical SP zone and thalamic reticular nucleus. (a) Corticofugal (blue) and thalamocortical (red) axons extend towards each other at early stages during embryonic development and reach close to their targets. However, they both stop short of their ultimate targets and corticofugal projections from subplate and layer VI accumulate in the thalamic reticular nucleus (TRN) and thalamocortical projections in subplate, respectively. Both compartments contain largely transient cells that get integrated into circuits during these early stages. (b) Towards the middle of the first postnatal week corticofugal and corticopetal axons enter the thalamus (Th) and cortical plate (CP), respectively, where they arborize and establish their contacts with their ultimate targets in thalamus and neocortex. There are signs of fibre decussations in the TRN and in the subplate indicating some rearrangements during development. Pale blue areas (amygdala, subplate and thalamic reticular nucleus) represent brain regions sharing gene expression patterns. Ag: amygdala; Hp: hippocampus; MGE: medial ganglionic eminence; MZ: marginal zone (green area); S: striatum. Figure from Montiel et al. (2011).

Ray was also interested in the rearrangements of the early corticofugal projections closer to the thalamus (Adams, Lozsádi & Guillery, 1997; Lozsádi, Gonzalez‐Soriano & Guillery, 1996; Mitrofanis & Guillery, 1993). He suspected similar fibre rearrangements occurred close to the thalamus. Our idea was that subplate is driving the rearrangement of thalamocortical projections, whilst the thalamic reticular nucleus has a similar role in the corticothalamic projections (Montiel et al., 2011). Interestingly, both subplate and thalamic reticular nucleus contain an early generated and largely transient population of cells with shared gene expression profiles (Figure 1; Wang et al., 2011; Montiel et al., 2011; Oeschger et al., 2012). Ray argued that these rearrangements must have occurred during development when these largely transient neurons become integrated into their circuits (Adams et al., 1997; Lozsádi et al., 1996; Mitrofanis & Guillery, 1993).

Ray once drew my attention to an interesting essay called “Mälzel's Chess Player” (1836) by Edgar Allan Poe (Poe, 1836) telling the story of an automaton chess player called “The Turk” which had become famous and toured widely in Europe and United States. The automaton was invented by Wolfgang von Kempelen in 1769 and, after von Kempelen's death, was brought to the United States in 1825 by Johann Nepomuk Mälzel. However, Mäzel's Chess‐Player was a fake and there was a chess playing expert hiding inside. Ray suggested that this is how we should start thinking about the subplate and thalamic reticular nucleus, little hidden men who sort out connections in the developing brain. Indeed, early work, including my own with Colin Blakemore, showed that the early connections were orchestrated by long‐range projections from subplate and layer 6b (Blakemore & Molnár, 1990; McConnell, Ghosh & Shatz, 1989; Molnár & Blakemore, 1995; Shatz et al., 1990). Numerous mutants later demonstrated that without these early projections, the thalamocortical fibres could not cross the pallial‐subpallial boundary and the co‐fasciculation of these fibres could explain the abnormal fasciculation patterns mutant “reeler” mice (López‐Bendito & Molnár, 2003; Molnár & Blakemore, 1995; Molnár, Adams, Goffinet, et al., 1998). Moreover, others and ourselves started to discover further transient circuits including GABAergic interneurons during cortical assembly (Marques‐Smith et al., 2016; Tuncdemir et al., 2016). Ray's idea was that “latticework”: arrangement of fibres close to the perireticular and thalamic reticular nuclei (PRN and TRN, respectively) might be involved in sorting fibres towards the appropriate thalamic nuclei in a similar way. John Mitrofanis had some interesting developmental evidence to support this idea, but we had to wait two decades before the various corticothalamic pathways from different subtypes of layer 6 and layer 5 could be examined in transgenic mice showing the detail of these rearrangements. The development and plasticity of this pathway are still not fully understood, but Ray followed and proofread some of the recent papers from my group on this topic (Grant, Hoerder‐Suabedissen & Molnár, 2012, 2016; Hoerder‐Suabedissen, Hayashi et al., 2018; Hoerder‐Suabedissen, Korrell et al., 2018).

We have now made great progress on the molecular taxonomy of the subplate neurons and their remnants in layer 6b, the transient neurons of thalamic reticular and perireticular (TRN/PRN) nuclei and also on the kinetics and transformations of representations between the thalamus and the cortex.

It is astonishing to see just how deep Ray's insights were in predicting developmental mechanisms that were not yet discovered. He knew the adult arrangements, he identified the locations of possible transformations of the representations, and then noticed that during development, these regions contained largely transient cell populations and, based on this, was able to predict the role they played during development. We still follow this intellectual framework to investigate thalamocortical development and plasticity. Ray attended some of our laboratory meetings after his return from Istanbul and followed this work in my laboratory with great interest and even asked to look at the original slides for some of the layers 5 and 6 and 6b reporter lines we have examined over the last few years (Grant et al., 2012, 2016; Horder‐Suabedissen & Molnár, 2013; Hoerder‐Suabedissen, Hayashi et al., 2018; Hoerder‐Suabedissen, Korrell et al., 2018).

## Teaching neuroanatomy with ray: “Structures with no known function”

After Ray returned from Department of Anatomy at Marmara University, in Istanbul, he volunteered to help us to run the medics neuroanatomy practicals on Monday mornings. We had a shortage of qualified staff and his help was very much appreciated in running the classes together with Jo Begbie, Jeremy Taylor and John Morris. Ray did it for several years until it became too tiring for him. The sessions are run in small groups in the dissection room and we have to repeat some sessions 4–6 times in the course of a morning. He often criticized us saying, “you do not teach them how to think.” Initially, I did not understand his disapproval because the classes included lots of clinical cases and clinical problem‐solving exercises that are designed to help medical students to learn exactly how to think about clinical syndromes, how to identify possible locations of lesions just by knowing the function and the location of nuclei, pathways, vasculature and their functions. The goal is to teach them how to employ their knowledge of neuroanatomy in the clinical diagnosis and management—that is how to think. However, in the neuroanatomy practicals, we taught them how to think as a future clinician. What Ray wanted us to do was not only teach the students what we know, but also teach them what we currently do not know. When a second year medical student would approach and ask Ray where the claustrum was, he would show them. But when they asked Ray about the function, he would just say: “we do not know.” This is not what medical students like to hear. He urged us to expose just how little we knew about the function of certain structures in the brain. Indeed, the deeper learning that we advocate during 3rd year (Final Honors School) could be started much earlier. In fact, the course for medicine at Oxford is moving in this very direction (Chang & Molnár, 2015).

## History of neuroscience and Ray

Whilst Ray was interested in the history of neuroscience, he once told me that he still wanted to focus on the present and future and perhaps at a later stage deal with history. Nevertheless, Richard Boyd and I managed to convince him to give a lecture on “The Visual Pathways in History: Maps of the World in the Brain” on 1 December 2011 (this is recorded and available on https://history.medsci.ox.ac.uk/seminars/history-of-medical-sciences-seminar-series/prof-ray-guillery-the-visual-pathways-in-history-maps-of-the-world-in-the-brain/ our History of Medical Sciences Project Website. This lecture is a superb illustration of how Ray integrated the old observations with the very new mechanistic research.

Ray also published a paper on the history of neuroscience in his later years, about Otto Friedrich Karl Deiters (1834–1863) (Deiters & Guillery, 2013). Ray had also noticed that I show the original Deiters pictures of bovine ventral horn motor neurons in my introductory lecture on cells of the nervous system. Ray was related to Deiters and he knew a great deal from family history about his work and life. Deiters did not have an easy life and Ray admired Deiter's dedication and drive. He was the second of five surviving children in an academic family. By a very young age, he had produced a highly original study of the brainstem and spinal cord. Ray wrote “He showed that most nerve cells have a single axon and several dendrites; he recognized the possibility that nerve cells might be functionally polarized and produced the first illustrations of synaptic inputs to dendrites from what he termed a second system of nerve fibers” (Deiters & Guillery, 2013). He had great academic promise, teaching and doing his research in the Bonn Anatomy Department whilst also running a small private practice and with an appointment in the University Clinic. However, when his father died, Deiters was left with his older brother responsible for supporting the entire family. But even in these difficult circumstances, he continued his work, and Ray was very impressed by Deiters’ dedication to research. He had a family to feed and struggled to get academic promotion, yet he had exceptional insight and produced some of the best observations at the time (Deiters & Guillery, 2013). He was never recognized during his life and his greatest scientific achievements were published posthumously. Otto Deiters eventually died of typhus in 1863 aged only 29.

## Cortical layer with no known function

**Figure 2:**
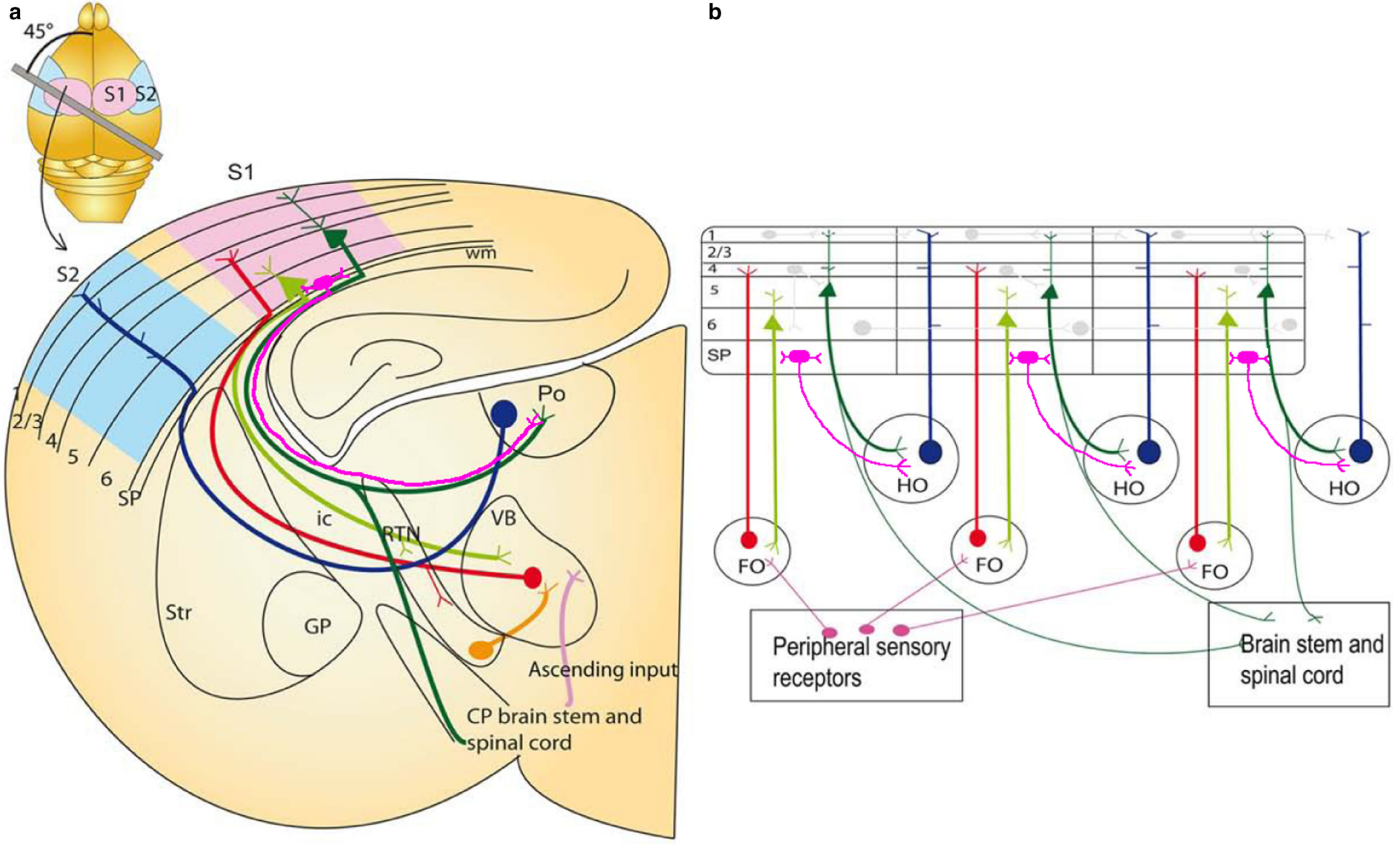
Thalamocortical circuits in the adult on an idealized section containing somatosensory cortical connections (a) and schematic representation of the two major sets of thalamic projection neurons (b). (a) Inset: outline of the mouse brain with the line indicating the plane of section to obtain thalamocortical slice containing S1 with intact thalamocortical projections. For clarity, S2 cortex connectivity is also indicated in the idealized section, although a different plane of section would be required to maintain connections. Main image: coronal schematic demonstrating the specificity of the connections between the cortex and thalamus using the somatosensory system as an example. The first order VB thalamic nucleus receives somatosensory peripheral input (pink). The VB then projects axons (red) to layer 4 of the primary somatosensory cortex (S1; light blue). Layer 6 “modulator” neurons (light green) in S1 project back to the VB. Layer 5 neurons (dark green) in S1 project to subcerebral structures and make a collateral branch to a higher order thalamic nucleus, for example, posterior thalamic nucleus (Po). Some of the persistent subplate cells at the bottom of layer 6 (SP, bright pink) selectively project to higher order thalamic nucleus Po, without giving a collateral to thalamic reticular nucleus (RTN). The higher order nuclei then project (dark blue) to an area of cortex that is different from the one they received input from (for example S2; light pink). This projection pattern generates an open loop. (b) Schematic illustration of the possible functional circuits generated by this reoccurring open loop connectivity and how persistent subplate (SP) can regulate the transthalamic cortico‐cortical communication through their projections (bright pink) to higher order thalamic nuclei. Sensory information is relayed through the first order thalamic nucleus to the cortex (red). This cortical area then projects from layer 6 reciprocally back to the first order nucleus (light green). Each area is also non‐reciprocally connected to a higher order thalamic nucleus. The layer 5 input to the thalamus (dark green) is an “efference copy” of the layer 5 output to the motor system in the brainstem and spinal cord. This copy is forwarded to a higher cortical area (blue). Some persistent subplate (SP) in layer 6b selectively project to higher order thalamic nuclei and in a position to regulate the transthalamic cortico‐cortical communication. Direct cortico‐cortical connections are also depicted between cortical layers and cortical areas (pale grey lines). These circuits enable cortical areas to act with other cortical areas and motor apparatus in a coordinated manner. CP: cerebral peduncle; FO: first order thalamic nuclei; GP: globus pallidus; HO: higher order thalamic nuclei; ic: internal capsule; RTN: reticular thalamic nuclei; SP: subplate; Str: striatum; S1: primary somatosensory cortex; S2: secondary association somatosensory cortex; Po: posterior thalamic nuclei; VB: ventrobasal thalamic nuclei; wm: white matter. Figure is modified from Grant et al. (2012) that was inspired by Guillery and Sherman (2002) and modified based on the results of Hoerder‐Suabedissen, Hayashi et al., 2018.

Ray Guillery and Murray Sherman had a very influential and general theory of corticothalamic interactions, including the distinction between driver and modulator pathways and their functions (Sherman & Guillery, 1996, 2011; Figure 2). First‐order thalamic nuclei receive inputs largely from peripheral sense organs and subcortical structures, and relay this information to the cerebral cortex. Higher order nuclei relay information from one cortical area to another and may occasionally receive subcortical input (Groh et al., 2014). Layer 5 usually provides input to higher order thalamic nuclei, via axon collaterals of subcortical projections targeting the superior colliculus and spinal cord, amongst others (Deschênes, Bourassa & Pinault, 1994; Deschênes, Veinante, & Zhang, 1998). These are powerful and large, feed‐forward “driver” inputs for relay to other cortical areas (Groh, de Kock, Wimmer, Sakmann & Kuner, 2008; Reichova & Sherman, 2004; Rouiller & Welker, 2000). They may converge onto thalamic cells also receive driver input from subcortical structures (Groh et al., 2014), but they do not form collaterals or synapse in the thalamic reticular nucleus (Bourassa, Pinault & Deschênes, 1995). In contrast, layer 6a cells form axon collaterals and synapse with inhibitory neurons in the TRN (Lam & Sherman, 2010) and this has an impact on the frequency‐dependent modulation of thalamic function (Crandall, Cruikshank & Connors, 2015). Roullier 1995 highlights that usually L5 giant projections are to higher order nuclei, but ventral posterolateral nucleus of thalamus (VPLc) and dMGB also receive giant L5 terminals, yet are considered “first order” nuclei.

Over the last few years, I had extensive discussion with Ray on the roles of the corticothalamic projections in this context. My laboratory became interested in the differences between the upper and lower parts of layer 6. Layer 6b is very different from layer 6a. Layer 6b neurons have different connectivity, cell types and gene expression patterns (Hoerder‐Suabedissen & Molnár, 2015). The morphology of these layers is so different that even Cajal considered them as two separate layers, calling the bottom one layer 7 (Cajal, 1909). Anatomical and transcriptomic analyses have revealed clear distinctions between layers 6a and 6b (Belgard et al., 2011; Hoerder‐Suabedissen et al., 2009; Marx & Feldmeyer, 2013; Oeschger et al., 2012). Layer 6b neurons express high proportions of susceptibility genes linked to human cognitive disorders, and the distribution of interstitial white matter neurons is known to be altered in schizophrenia and autism (Akbarian et al., 1993; Bakken et al., 2016; Hoerder‐Suabedissen et al., 2013; Kostović, Judaš & Sedmak, 2011; Miller et al., 2014). In spite of these clinical links, our current knowledge about developing layer 6b is limited and even less is known about its function in the adult.

My laboratory and our collaborators are currently examining the input and output characteristics of these neurons using various tracing methods (Hoerder‐Suabedissen, Hayashi et al., 2018). Retrograde labelling studies targeting posteromedial and lateral posterior thalamic nuclei (PO and LP) have suggested that layer 6b might specifically target these nuclei from primary somatosensory cortex (S1) (Killackey & Sherman, 2003) and primary visual cortex (V1; Roth et al., 2016), but the injections were not precise enough to suggest lamina‐specific patterns.

We had many discussions with Ray about the possible function of these layer 6b projections to the higher order thalamic nuclei. Layer 6a projections have dynamic synapses in thalamus that depend upon their firing frequency (Crandall et al., 2015). Layer 6a projections stimulate the thalamic projection neurons directly and inhibit them through collaterals that stimulate the thalamic reticular nucleus. Depending to the balance of these influences, they modulate the sensory gating (Crandall et al., 2015). Layer 6b projections from Drd1a‐cre+ neurons do not have side branches or synapses in TRN so do not make connections with inhibitory neurons (Hoerder‐Suabedissen, Hayashi et al., 2018). These layer 6b projections could act as stimulatory counterparts of zona incerta projections that also selectively target the higher order thalamic nuclei (Mitrofanis, 2005). However, the layer 6b terminals in PO are always small in contrast to layer 5 projections, which can be large and small in the Rbp4‐cre line (Hoerder‐Suabedissen, Hayashi et al., 2018). This suggests that layer 6b projections might be involved in modulating rather than the driving of the higher order thalamic nuclei. The higher order thalamic nuclei are involved in local cortical state control (Schmitt et al., 2017). Layer 6b projections to the cortex and to the higher order thalamic nuclei could form the anatomical substrate of the pathways that regulate which part of the thalamocortical circuits should be active and how transthalamic cortico‐cortical communication is regulated. These modulatory projections to the higher order thalamic nuclei could be particularly important when there is contextual conflict and more attention should be paid to a particular sensory input. For example, if we suddenly realize that something is “just not right” or “novel” in our environment, we have to adjust the sensory gain to get better, more precise information. Thus, layer 6b projections and contacts with thalamic cells may open up transthalamic cortico‐cortical communications especially through the cortical areas involved in higher cognitive processing. These projections to the higher order thalamic nuclei might enable processing of sensory input in a more global context (Guidi et al., 2016). The layer 6b projections to higher order thalamic nuclei could be involved in local and global cortical state control. A subset of layer 6b cells are intrinsically bursting, responsive to neurotensin, dopamine, histamine and noradrenaline, have a spiny, non‐pyramidal shape, and are depolarized by orexin in a manner that stops intrinsic bursting (Bayer et al., 2004). It is of significance that both layer 6b neurons and their targets, the higher order thalamic nuclei, are selectively sensitive to orexin (Bayer et al., 2004; Hay, Andjelic, Badr & Lambolez, 2015). This part of the thalamus falls asleep first when we go to sleep. Ray followed these developments with great interest and we had long debates about these findings with Anna Hoerder‐Saubedissen, Elenor Grant, André Marques‐Smith and Shuichi Hayashi.

## Founding the thalamus club

The Cortex Club was established in May 2009 by a group of Oxford neuroscience DPhil students spearheaded by Abhishek Banerjee and Dennis Kaetzel and registered with the Proctors of University of Oxford under my sponsorship as senior member in December 2009. It maintains an official university club status. It has a committee elected from graduate students and postdocs. However, it is open to the entire academic community from undergraduates to professors and attracts large and enthusiastic audiences. The Cortex Club grew into a unique forum dealing with cutting‐edge topics and significant challenges in neuroscience. The events range from small intense debates with up‐and‐coming scientists, to large discussion sessions led by internationally prominent speakers, followed by the opportunity for the students to questions over drinks (https://cortexclub.com/about/). Ray attended Cortex Club meetings very regularly and also spoke at them—once on his own and once with Murray Sherman. He was very fond of this organization. But he also wanted to establish the “Thalamus Club,” as a smaller and even more spontaneous and informal group than the Cortex Club. He started gatherings in the small, windowless room within the Lamb and Flag pub. Ray found it appropriate that the Thalamus Club (thalamus—means inner chamber) met in this small inner room. We had several sessions with Ray, and the discussions were very stimulating. We even had our own Facebook page: https://www.facebook.com/ThalamusClubOxford/. The Thalamus Club continues in the spirit of Ray's original vision and its current president, Dr Kouichi C Nakamura, recently arranged sessions to discuss Ray's book, “The Brain as a Tool” (2017, Oxford University Press).

It was Ray's wish to donate his personal book collection to the Department of Physiology, Anatomy and Genetics. After Ray's death, I arranged a meeting with Ray's son Peter and I packed up most of Ray's books and delivered them to the Sherrington Library. Peter and his son dealt with the rest of the house. Whilst I was packing the books in Ray's house, I noticed just how many books he had on how to write well, how to use grammar, and how to pass on a scientific concept. This made me realize that I should have worked much harder on my academic writing during my early years. Ray helped me and all his other seedlings and colleagues in UK, China, Turkey and other countries to write papers and grants (Onat, Oğlu & Çavdar, 2018 in this issue). I always considered him as someone who had this gift of writing effortlessly. The books I found in his house suggested that he checked and pondered on the best possible way to express his thoughts. His command of English was superb, but he did not take this for granted and he seems to have consulted things regularly using these reference books. Ray passed his knowledge and wisdom on how to write papers to us all by commenting on papers and by writing essays for the next generation (see Guillery, 2008a, 2008b).

Ray's spirit is with us through the science he produced, through the intellectual milieu he created. Now, when we discuss some new findings on thalamocortical interactions, we often wonder what Ray would think and say and what further experiments he would recommend. I consider myself very fortunate to have known him.
